# Applying resource bricolage theory to the city integration of new-generation migrant workers in China

**DOI:** 10.1371/journal.pone.0256332

**Published:** 2021-08-20

**Authors:** Ai-xiang Zheng

**Affiliations:** 1 School of Government, Nanjing University, Nanjing, Jiangsu, China; 2 School of Management, Wuxi Institute of Technology, Wuxi, Jiangsu, China; Institute for Advanced Sustainability Studies, GERMANY

## Abstract

New-generation migrant workers in Chinese cities are struggling with a lack of urban resources, such as capital, skills, and relationships. To cope with the pressure of these resource constraints, new-generation migrant workers obtain urban development opportunities through resource bricolage. Based on a questionnaire survey of 365 new-generation migrant workers, we used a multiple regression analysis to study the mechanism underlying the effects of resource bricolage on the city integration of new-generation migrant workers. There were four findings: (1) resource bricolage had a significant positive effect on career growth and city integration; (2) career growth had a mediation effect on the relationship between resource bricolage and city integration; (3) environmental dynamism had a positive moderating effect on the relationship between resource bricolage and city integration for new-generation migrant workers; and (4) resource bricolage and environmental dynamism had a moderating effect on city integration through the mediation effect of career growth. The results suggest that resource bricolage promotes the career growth of new-generation migrant workers and further promotes their city integration, and that the environmental dynamism faced by workers is the external condition for promoting integration through resource bricolage. The study emphasizes the importance of resource bricolage in new-generation migrant workers’ city integration.

## Introduction

Migrant workers are a unique group in contemporary Chinese economic and social development. They work in cities when farm work is scarce and return to their hometowns to engage in agricultural production when needed. According to statistics from the National Bureau of Statistics of China, there are currently 287 million domestic migrant workers in China. Among this group, migrant workers born in 1980 and later are called new-generation migrant workers, of which the total population has reached 145 million [1]. Compared with the previous generation of migrant workers, this group has both commonalities and differences. Although their vocational skills are not as strong as those of the previous generation of migrant workers, they have a stronger desire to become citizens and prefer to stay in the city instead of returning to the countryside [2]. However, although new-generation migrant workers have a strong desire to become citizens and can achieve basic urban employment, most of them experience poor working conditions and low wages and find it difficult to truly integrate into urban society [3].

The issue of how to continuously promote the urban integration and citizenship of new-generation migrant workers is a major concern in Chinese academic circles and among all levels of government. Logically, increasing the urban resources held by new-generation migrant workers is a key step towards ensuring and promoting their urban integration.

At present, however, many new-generation migrant workers face a lack of urban resources, such as capital, skills, and relationships. Due to this lack of resources, the challenges faced by new-generation migrant workers in cities are much greater than those of other urban populations. Therefore, resource issues can be considered one of the main obstacles to the flourishing of new-generation migrant workers in urban areas.

Resource bricolage theory opens a theoretical window on this issue. Resource bricolage refers to how entrepreneurs seek and combine the resources they have at hand under the condition of resource constraints. The theory predicts that the seeking, “making do,” and combining of the resources at hand through resource bricolage will facilitate the discovery of new opportunities and realization of new values [4].

In terms of entrepreneurs, new-generation migrant workers have the attributes of quasi-startups. They are thus characterized by “weakness” in two respects [5,6]. First, in the process of urban survival and flourishing, they face the same challenge as that faced by start-ups, which is a lack of resources. Second, similar to the entrepreneurial vision of a start-up, they have the goals of capital accumulation from scratch and of improving their quality of life.

Considering that the circumstances and purposes of new-generation migrant workers in urban settings are highly compatible with those of the entrepreneurs theorized as engaging in resource bricolage, this article applies resource bricolage theory to an examination of the mechanisms of city integration among new-generation migrant workers. This will not only help to further explore ways to promote city integration of new-generation migrant workers, but can also expand the scope of the application of resource bricolage theory. The study therefore has theoretical and practical significance for exploring the urban integration of new-generation migrant workers in China.

## Literature review

### Research on the urban integration of migrant workers

A substantive sign of citizenization is the degree of urban integration of the agricultural migrant population with respect to occupation, income and psychology [7]. New-generation migrant workers are an important component of the overall agricultural migrant population. How to continuously promote their urban integration and help them to achieve citizenship is a concern of academics and governments. Previous research has mainly focused on policies of urban integration, especially at the macro level of household registration (Hukou) reform and healthcare provision. Wang argued that holistic social policy reform and innovation are needed to promote social integration [7]. Qin and Li pointed to the urban residence status, employment conditions, and urban medical insurance as important influences on the urban integration of migrant workers [8]. As difficulties in housing and employment are gradually eliminated, medical insurance is becoming increasingly important as an issue in urban integration.

In recent years, some scholars have begun to shift to a micro perspective on the new-generation migrant workers’ personal factors. Zhang [9] and Zhao [10], for example, both claimed that the level of human capital investment would affect the willingness of new-generation migrant workers to remain in a city, and that human capital is the basic condition for ensuring the integration of new-generation migrant workers. Meanwhile, Zhang and Li [11] and Liu and Wong [12] identified social capital as a foundation for the survival and development of new-generation migrant workers in cities. Most migrant workers obtain employment and other opportunities in their host cities through personal relationships. Despite these studies of the effects of human capital and social capital in the integration of new-generation migrant workers, there has been little research on the roles of different types of resources in integration.

### Resource bricolage theory and its application to agricultural workers

Resource bricolage theory is mainly focused on entrepreneurship. Shane and Cable [5] and Baker and Nelson [4] found that most entrepreneurs face the problems of information asymmetry and opacity in their business operations. Therefore, it is often difficult for them to obtain external support, and the resources available for their disposal are very scarce [4,5]. With new ventures facing the reality of resource constraints, Baker and Nelson put forward the notion of resource bricolage, which involves seeking, making do, and combining those resources that are at hand but have not yet been properly valued or utilized [4]. Resource bricolage helps companies to adapt to environmental pressures, obtain human capital and ready-to-hand capabilities [13,14], improve entrepreneurial performance [15,16], and expand organizational influence and competitive advantage [17,18].

In recent years, some Chinese domestic scholars have applied resource bricolage theory to research on agricultural workers. Gao and Zhang found that selective resource bricolage strategies helped farmers to realize their entrepreneurial value [19]. Zhang et al. summarized the resource types and specific strategies of resource bricolage among farmer entrepreneurs [20]. From the perspective of migrant workers’ entrepreneurship, Luo analyzed the types of resource bricolage used by migrant workers at different stages of citizenization [21]. Li et al. found that resource bricolage promoted the performance and sense of acquisition of family farms [22]. Despite having enabled these breakthroughs in the study of agriculture, resource bricolage theory has not been applied to the process of citizenization of new-generation migrant workers. From an entrepreneurial theory point of view, new-generation migrant workers have the attributes of quasi-startups and face a resource situation similar to that theorized by resource bricolage theory. New-generation migrant workers need to realize their own urban survival, integration, and flourishing by utilizing resources in a situation of resource constraints.

New-generation migrant workers have indeed applied the strategy of resource bricolage in their daily work and lives. However, little is known about the effects of the resource bricolage of new-generation migrant workers on their citizenization and urban integration. This paper expands the theoretical application of resource bricolage theory to the effects of resource bricolage on the integration of new-generation migrant workers in cities.

## Theoretical frameworks and research hypotheses

### The influence of resource bricolage on urban integration

Compared with the local urban labor force, new-generation migrant workers have long been in a weaker position due not only to lower levels of education but also to a severe lack of urban resources, such as interpersonal networks, housing, and financial support from family [11,23]. To cope with the pressure of resource constraints, some new-generation migrant workers take a bricolage approach to the problem of resource scarcity: “seeking” the existing resources at hand and carrying out resource “making do” and “combining.” Such resource bricolage not only plays a role in the pre-citizenization stage of new-generation migrant workers but also runs through the entire process of citizenization. This is not only related to the long-term relative shortage of urban resources for new-generation migrant workers but also to their goals of breaking through resource constraints and adapting to the needs of long-term career growth in the process of citizenization.

In the pre-citizenization stage, resource bricolage among new-generation migrant workers mainly involves the use of their original rural resources. Deng [24] and Wang and Wu [25] argued that migrant workers achieve employment mainly by combining and utilizing their rural relationship resources, and they found that this was extremely common in the manufacturing and construction industries, where most of the new-generation migrant workers in our survey were working.

After new-generation migrant workers achieve employment, gaps in their individual career growth and urban integration gradually open up between them [25]. The individual resources of new-generation migrant workers have the most obvious effects on their development. New-generation migrant workers who are good at resource bricolage are more adaptable to the urban working and living environment in this period, meaning that the content of their resource bricolage begins to evolve from an early reliance on rural resources to a reliance on urban resources and even a mixture of urban and rural resources [26].

In terms of the specific types of resources involved in bricolage, in addition to their traditional rural relationships and physical resources, the experience resources and technical resources formed before and after entering the city form part of the combining resources of new-generation migrant workers [24,27]. Li and Li found that new-generation migrant workers in the decorating industry who were able to seek and combine urban and rural resources had a much better urban career growth trajectory than those who relied only on rural resources [28]. These successful new-generation migrant workers were both decorators and businessmen. They drew not only on their decorating experience and skills but also on the resources of fellow villagers to form temporary decorating teams. They also had the ability to develop certain local urban resources and to combine experience resources, urban labor and customer resources, technical resources, and physical resources [25]. This resource bricolage was closely related to their successful urban integration.

Ren et al. [29] and Xu [30] stated their belief that through the combination of certain types of resources, such as relationships and technology, the gap in urban resources between new-generation migrant workers and local citizens can be continuously reduced, and the career growth and quality of urban life experienced by migrant workers can approach or even exceed that of locals. Based on the above analysis, resource bricolage by new-generation migrant workers is closely related to their career growth and urban integration. In view of this, the following hypotheses are proposed:

Hypothesis 1: Resource bricolage has a significant positive effect on the urban integration of new-generation migrant workers.
Hypothesis 2: Resource bricolage has a significant positive effect on the career growth of new-generation migrant workers.

### The influence of career growth on urban integration

Career growth refers to the career progress of an individual at a specific point in time [23,31], including the development and improvement of skills, knowledge, and experience [23]. Career growth among new-generation migrant workers has certain group traits that need to be observed as part of the general process of citizenization. Career growth among new-generation migrant workers is inseparable from the development of individual citizenship and is embedded in their citizenship process. The career growth of new-generation migrant workers can be considered as the upward and positive movements in the process of citizenization related to their careers, including progress toward career goals, salary growth, vocational skills improvement, and promotion speed, in the material and psychological domains [23].

From the perspective of the logical relationship between career growth and urban integration, career growth has a fundamental role in the process of citizenization and is an important prerequisite for the substantive promotion of material and psychological integration [32]. Furthermore, if new-generation migrant workers want to integrate into cities and become new citizens, continuous and stable career growth is needed because it is difficult to maintain citizenization without the support of career success. In material terms, career growth can provide financial support to new-generation migrant workers to enable integration into the city. The level of financial support directly determines the possibility of urban integration and the level of integration that can be achieved. In psychological terms, career growth can also provide the motivation for new-generation migrant workers to integrate into the city, because career successes such as promotion or skill improvement can provide a sense of happiness and accomplishment [33], which is a necessary condition for psychological integration. In short, career growth provides the impetus for urban integration. The career growth of new-generation migrant workers is essential to their personal development and thus to their integration into the city. In view of this, the following hypothesis is proposed:

Hypothesis 3: Career growth has a significant positive effect on the urban integration of new-generation migrant workers.

### The mediating effect of career growth

Career growth is not automatic, being the combined effect of an individual’s innate endowments and acquired investments [23]. Zuo and Sun [34] and Huang and Li [35] pointed out that new-generation migrant workers promote their own career growth through their behavior. As part of optimizing their human capital, migrant workers can accumulate and increase their own resources of skill and creativity [34,35]. This is the force driving the career growth of new migrant workers, and it ultimately promotes their realization of citizenship.

Some new-generation migrant workers perform resource making do and combination on the basis of seeking resources and maximize the efficiency of resources at hand through the structural and functional reorganization of resources. According to a case from Luo [21], one new-generation migrant worker interviewed said that a few years ago, after working in various occupations, he raised funds and invited his former colleagues to jointly open a metal processing workshop. Members of his family also joined and worked at the workshop. Interacting with customers and local workers has become his daily work, and he now earns an income incomparably higher than that he earned when he first entered the city. His career success brought continuing improvements and changes to his city life. And now he bought an apartment and a car by himself, and his achievement got the praise of locals. This is an example of how the resource bricolage of new-generation migrant workers can affect individual career growth and city integration.

In summary, the resource bricolage of new-generation migrant workers can provide resources and capabilities for their career growth, which in turn promotes their urban integration. Career growth has certain connecting and transformational functions in the relationship between resource bricolage and city integration, and it is the key hub and node in this mechanism. The citizenization of new-generation migrant workers is an important part of the new urbanization in China. In the process of China’s new urbanization, career growth is the foundation and process goal of the flourishing of new-generation migrant workers in the cities. With the help of continuous career growth, the integration of these workers into the city is achievable as the fundamental goal of urban development. In view of this, the following hypothesis is proposed:

Hypothesis 4: Career growth has a mediation effect on the relationship between resource bricolage and urban integration among new-generation migrant workers.

### The moderating effect of environmental dynamism

Environmental dynamism reflects the speed and degree of changes in the external environment [36]. It is an important variable in the behavior of economic entities. Environmental dynamism includes not only the technological dynamics of technological substitution and upgrading but also the market dynamics caused by changes in market demand and increased competition [37]. Environmental dynamism has mostly been studied by business scholars, who generally hold that environmental dynamism brings challenges but also opportunities, which are the driving force of corporate innovation [38]. In fact, new-generation migrant workers are also positioned within this dynamic environment, and it therefore has an effect on the success of their integration into the host city.

Compared with the rural environment, the urban environment is more competitive and the pace of change is more rapid. How to adapt to such a dynamic environment is the primary challenge faced by new-generation migrant workers in their urban lives [39]. More importantly, new-generation migrant workers also face long-term challenges from the dynamics of the market and technological environment. These challenges run through the entire urban careers of new-generation migrant workers. Specifically, these workers must continuously improve their ability to adapt to the environment to meet their needs for career growth and urban integration. At the same time, environmental dynamism is the rich soil in which new-generation migrant workers can grow by absorbing new technologies and new knowledge, and technological and market dynamics drive career and development opportunities.

Environmental dynamism is closely related to resource bricolage, as individuals make do and combine the resources at hand in response to environmental changes [40]. Indeed, the combination of resources in the face of environmental pressure is the central feature of resource bricolage. In a dynamic environment, the desires to obtain career opportunities and achieve urban integration motivate new-generation migrant workers to seek and re-combine resources. Although this was a response of new-generation migrant workers to their external environment using only at-hand resources, it was highly effective. The dynamic external environment provides the external conditions and opportunities for new-generation migrant workers to combine resources [41]. The current highly dynamic environment is constantly squeezing their living space and opportunities in cities [24]. To gain a foothold in the city, new-generation migrant workers need to continuously expand the scope and increase the means of resource bricolage. In view of this, the following hypothesis is proposed:

Hypothesis 5: Environmental dynamism has a positive moderating effect on the relationship between resource bricolage and new-generation migrant workers’ urban integration.

The impact of environmental dynamism on new-generation migrant workers is first directly reflected in the impact on their career [42,43]. One taxi driver who entered the city from the countryside told us that the taxi business had been declining in recent years [44]. They were not only facing increased competition from additional private cars but also from the arrival of online taxi services. In response, the driver had established a WeChat group with several other drivers to share the customer demand. He said that if they notice that there are orders nearby, they exchange this information through the chat group, which has improved the efficiency of receiving orders and increased their income. Their taxi network is continuing to expand, and some other taxi drivers from the same company have also joined.

This is an example of how the dynamics of the external environment encourage new-generation migrant workers to make do and combine technology and network resources. The impact of environmental dynamism on urban integration of new-generation migrant workers is in fact influenced through their career growth. That is, the more dynamic the environment, the greater the impact of resource bricolage on the career growth of new-generation migrant workers, and the correspondingly greater the impact on the urban integration.

It is clear that the impact of environmental dynamism complicates the mechanism through which resource bricolage affects urban integration. The moderating effect of environmental dynamism on the relationship between resource bricolage and urban integration works through the transformational mechanism of career growth. In view of this, the following hypothesis are proposed:

Hypothesis 6: The moderating effect of resource bricolage and environmental dynamism affects urban integration through the mediation effect of career growth.

**Fig 1:**
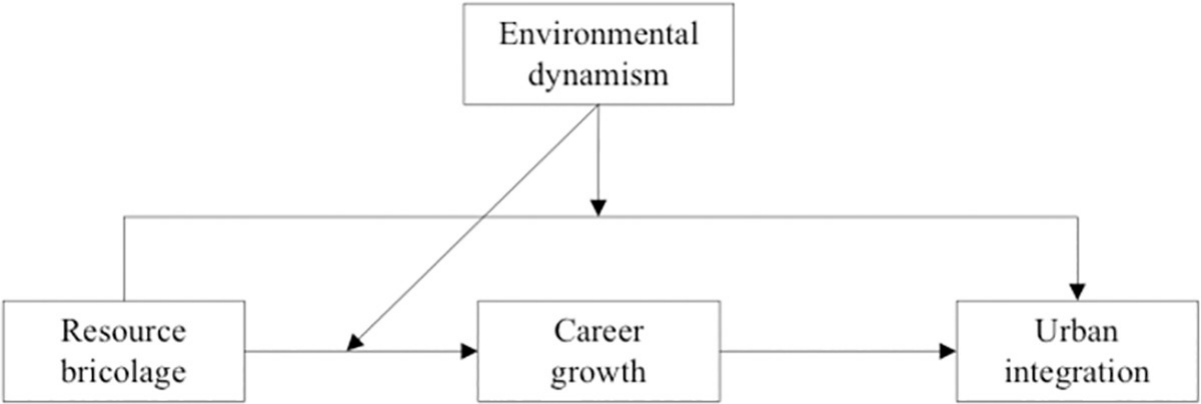
Theoretical model of the mechanisms of resource bricolage on urban integration.

In summary, the theoretical model constructed in this paper is shown in Fig 1.

## Materials and methods

### Variable measurement

The scale for the survey used in this study was mainly adapted from domestic and foreign sources. A 5-point Likert scale was used for all items, ranging from 1 (*strongly disagree*) to 5 (*strongly agree*).

To improve the reliability and validity of the scale for the main study, the initial formulation of the scale was optimized through expert review and pre-testing. We first invited professors in the field to review the form and content of the scale. The invited experts made suggestions on the order of the items and the appropriateness and accuracy of the expression of the items. After making revisions based on this feedback, we invited 14 new-generation migrant workers to participate in a pre-test, which further confirmed the legibility and accuracy of the questionnaire. After these two rounds of optimization, a formal scale was formed for use in the main study. The final scale variables were as follows:

1. *Resource bricolage* was measured using eight items based on the scale of Senyard et al. [45]. Representative items are “I am confident of our ability to find workable solutions to new challenges by using our existing resources,” “I gladly take on a broader range of challenges than others with our resources would be able to,” and “I use any existing resource that seems useful to responding to a new problem or opportunity.”
2. *Career growth* was measured using 15 items drawing on the scale of [42], with items addressing the progress of career goals and the speed of promotion and increases in compensation. Representative items are “My present job moves me closer to my career goals,” “My present job encourages me to continuously gain new job-related skills,” and “My salary is growing quickly in my present organisation.”
3. *Environmental dynamism* was measured using four items drawing on the scale of Li et al. [46]. Representative items are “Products or services in our industry update quickly,” “The acts of competitors are difficult to predict,” and “The technology in our industry progresses quickly.”
4. *Urban integration* was measured using 15 items referring to the scale of Luo et al. [47]. Representative items are “I think I am a local now,” “My current income can satisfy my needs in my local life,” and “My local circle of friends includes mostly local people.”

To enhance the completeness and interpretability of the model, we adopted the variable design suggestions of Liu [48] and Qian et al. [49] in adding the individual characteristics of gender, age, and educational background as control variables in the measurement model.

The ethical aspects of this study complied with the ethical guidelines for scientific research of Wuxi Institute of Technology, and the study was approved by the Review Board at Wuxi Institute of Technology on 22 October, 2020. Prior to the survey, an informed consent form was issued to the participants, including information on the objectives and methods of the study and the participants’ time demands and rights. All participants were required to sign this informed consent form before proceeding with the questionnaire. All questionnaires were completed anonymously. Furthermore, to improve the quality of the questionnaire answers and dispel any confidentiality concerns of the respondents, the questionnaire instructions stated “We will keep your responses confidential, and the survey results will only be used for scientific research purposes” and “There is no correct answer to any item. Please fill in the information according to your actual situation.”

### Research methods and data collection

Empirical research methods were used to study the mechanisms of the effects of resource bricolage on urban integration. In the research process, the researchers used SPSS (version 19) and AMOS (version 18) to measure the reliability and validity of the model, and on this basis, tested the relationships between the variables in the model to verify the hypotheses. Research conclusions were then drawn from the statistical results.

The survey was conducted in November 2020. A stratified random sampling method was adopted, with most participants working in Jiangsu, Zhejiang, and Shanghai in the Yangtze River Delta region of China. In setting the criteria for new-generation migrant workers, we drew on the *National Migrant Workers Monitoring Survey Report* of the National Bureau of Statistics and the definitions of domestic scholars [20,48], who generally agree that migrant workers born after 1980 are to be considered part of the “new generation.”

Regarding the number of questionnaires issued, Hair suggested that to ensure quality, this number should be 5–20 times the total number of items on the scale [50]. With reference to this standard, 332 questionnaires were distributed, of which 327 were recovered. After the questionnaires were collected, the quality of the answers was checked immediately. After inspection, it was found that 18 of the questionnaires were invalid, and these were eliminated from the analysis. A total of 309 valid questionnaires were returned, yielding a valid questionnaire ratio of 94.5%.

## Data analysis and results

### Descriptive statistical analysis

Table 1 presents the demographic characteristics of the sample. Men and women accounted for 68.9% and 31.1% of the sample, respectively. The breakdown of age groups was as follows: 7.8%, 23.0%, 17.8%, 19.1%, 27.2%, and 5.2% of respondents were aged 16–20, 21–25, 26–30, 31–35, 36–40, and 41–42 years, respectively. One per cent had an education level of primary school and below, 23% had completed middle school, 50.2% had completed high school, 24.3% had a college education, and 1.6% had post-college education. Workers with monthly incomes of less than 2,000, 2,000–3000, 3,000–4000, 4,000–5,000, 5,000–6,000, 6,000–7,000, 7,000–8,000, 8,000–9,000, and above 9,000 yuan accounted for 3.6%, 12.0%, 23.9%, 20.4%, 20.4%, 11.7%, 4.5%, 3.6%, and 3.3% of the sample, respectively. Most of the workers (83.2%) were married, with unmarried participants accounting for 14.2% and divorced participants for 2.6% of the sample. Based on these demographics, the main indicators of gender, education level and marital status in the sample are similar to those in the *National Migrant Workers Monitoring Survey Report 2019*, which shows that the selected sample was strongly representative.

**Table 1:** Sample characteristics *(N = 309)*.

	Classification	*n*	Percentage		Classification	*n*	Percentage
**Gender**	Male	213	68.9	**Monthly income (yuan)**	Below 2,000	11	3.6
	Female	96	31.1		2,000–3,000	37	12.0
**Age group (years)**	16–20	24	7.8		3,000–4,000	74	23.9
	21–25	71	23.0		4,000–5,000	63	20.4
	26–30	55	17.8		5,000–6,000	63	20.4
	31–35	59	19.1		6,000–7,000	36	11.7
	36–40	84	27.2		7,000–8,000	14	4.5
	40–41	16	5.2		8,000–9,000	11	3.6
**Education level (highest level completed)**	Primary school and below	3	1.0		9,000 or above	12	3.3
	Middle school	71	23.0	**Marital status**	Married	257	83.2
	High school	155	50.2		Unmarried	44	14.2
	College	75	24.3		Divorced	8	2.6
	Above college	5	1.6		Total	309	100%

Table 2 shows the descriptive statistics and correlation coefficients for each variable in the study. Resource bricolage, career growth, environmental dynamism, and urban integration had significant correlations and warranted further analysis.

**Table 2:** Means, standard deviations, and correlation matrix.

Variable	Mean	Standard deviation	Gender	Age	Education level	Resource bricolage	Career growth	Environmental dynamism	Urban integration
Gender	0.689	0.464	1	—	—	—	—	—	—
Age	3.504	1.427	0.194**	1	—	—	—	—	—
Education level	3.025	0.76	−0.208**	−0.362**	1	—	—	—	—
Resource bricolage	3.547	0.555	−0.195**	−0.108	0.013	(0.822)	—	—	—
Career growth	3.288	0.607	−0.059	−0.052	−0.035	0.592**	(0.856)	—	—
Environmental dynamism	3.521	0.593	−0.020	0.037	−0.087	0.527**	0.576**	(0.720)	—
Urban integration	3.475	0.493	0.102	0.238**	−0.104	0.344**	0.435**	0.363**	(0.740)

The diagonal of the correlation coefficient is the arithmetic square root of the AVE (average variance extracted).

* *p* < 0.05

** *p* < 0.01.

### Test for common method bias

Hair et al. believed that a large number of highly correlated variables is an initial indicator of potential common method bias [51]. However, they also pointed out that individual correlation coefficients below 0.8 are within an acceptable range [51]. In a further analysis of the correlation coefficients, it was found that the correlation coefficient of each variable in this study was far below this upper limit. Therefore, the problem of common method bias was preliminarily ruled out.

Considering that all of the questionnaire items in this study were answered by new-generation migrant workers themselves, there is still the possibility of common method bias. Therefore, to test more thoroughly for common method bias, this study adopted the suggestion of Podsakoff et al. in using Harman’s single factor test [52]. The basic assumption of this test method is that in the presence of homologous bias, there will be a single factor explaining most of the variance. We found that the first factor variance contribution rate in exploratory factor analysis was only 35.654%, which is well below the 50% standard threshold. This indicates that common method bias was not a major concern in this study.

### Reliability and validity analysis

A reliability analysis is used to measure the stability and credibility of measurement tools. As shown in Table 3, the Cronbach’s α values of the main variables in this study were 0.943, 0.956, 0.776, and 0.865, all of which are above the prescribed standard value. The corresponding combined reliability (CR) values were 0.943, 0.976, 0.798, and 0.947, respectively, all of which exceed the standard threshold of Fornell and Larcker [53]. This indicates that the reliability of the questionnaire is relatively high, and the items can explain the research variables consistently.

**Table 3:** Reliability and validity results.

Variable	CR	Cronbach’s α	KMO
Resource bricolage	0.943	0.943	0.929
Career growth	0.976	0.956	0.945
Environmental dynamism	0.798	0.776	0.749
Urban integration	0.947	0.865	0.860

CR = composite reliability; KMO = Kaiser–Meyer–Olkin value.

A validity analysis is used to verify that the measurement tool can accurately measure the correctness of the required variables. It is generally measured by the Kaiser–Meyer–Olkin (KMO) test and Bartlett’s test. As shown in Table 3, the KMO value of each variable was above the prescribed standard, and the concomitant probability of Bartlett’s sphericity test is 0.000, which indicates that the scale and its factors have high construct validity. Furthermore, the correlation coefficients of the main variables, as presented in Table 2, are all less than the arithmetic square root of their AVE values, indicating that the variables in this study have good discriminative validity.

## Results

### Test of main and mediation effects

This study followed the steps proposed by Baron and Kenny to conduct the main effect and mediation effect tests in sequence [54]. First, we carried out the main effect test of the independent variable (resource bricolage) on the dependent variable (urban integration). Model 1 in Table 4 shows the influences of control variables such as gender, age, and educational background on urban integration, and Model 2 shows that resource bricolage had a positive effect on urban integration (β = 0.397, *p* < 0.001). These findings support Hypothesis 1.

**Table 4:** Regression analysis results.

	Urban integration			Career growth		Urban integration		Urban integration		Career growth	Model
	Model 1	Model 2	Model 3		Model 4		Model 5	Model 6	Model 7	Model 8	Model 9
Gender	0.057	0.132**	0.053		0.085*		0.114*	0.118*	0.121*	0.034	0.112*
Age	0.223***	0.260***	−0.011		0.253***		0.264***	0.246***	0.255***	−0.023	0.261******
Education level	−0.011	0.013	−0.036		0.022		0.025	0.026	0.042	0.002	0.041
Resource bricolage		0.397***	0.602***				0.191**	0.275***	0.266***	0.393***	0.159*
Environmental dynamism								0.225***	0.191**	0.327***	0.102*
Resource bricolage × Environmental dynamism									0.139**	0.155**	0.097*
Career growth					0.453***		0.343***				0.272***
F	6.497	20.293	41.803		27.223		24.331	19.809	18.030	44.649	18.677
R^2^	0.060	0.211	0.355		0.264		0.286	0.246	0.264	0.470	0.303

* *p* < 0.1

** *p* < 0.01

*** *p* < 0.001.

Second, we tested the relationship between the independent variable (resource bricolage) and the mediation variable (career growth). Model 3 shows that resource bricolage had a positive effect on career growth (β = 0.602, *p* < 0.001), thus supporting Hypothesis 2. Third, we tested the relationship between career growth and urban integration. Model 4 shows that the β value of career growth in urban integration was 0.453 and significant at the level of p < 0.001, indicating support for Hypothesis 3.

Fourth, we tested the mediating effect of career growth on resource bricolage and urban integration. Model 5 shows that the effects of resource bricolage on career growth (β = 0.343, *p* < 0.001) and urban integration (β = 0.191, *p* = 0.002) were significant. The latter result was lower than the coefficient in Model 2, which shows that career growth had a partial mediation effect on the relationship between resource bricolage and urban integration. Hypothesis 4 was therefore supported.

### Mediated moderation effect test

We conducted the mediated moderation effect test according to the steps proposed by Wen and Hou [55]. Model 6 in Table 4 adds environmental dynamism as a moderating variable to Model 5 to test the effects of resource bricolage and environmental dynamism on urban integration. The results show that environmental dynamism was significantly related to urban integration (β = 0.225, *p* < 0.001).

On this basis, Model 7 tested the effects on urban integration of resource bricolage, environmental dynamism, and the interaction term of environmental dynamism and resource bricolage. The results show that the interaction term of resource bricolage and environmental dynamism had a significant effect on urban integration (β = 0.139, *p* = 0.008), supporting Hypothesis 5.

We subsequently tested the effects on career growth of resource bricolage, environmental dynamism, and the interaction term of environmental dynamism and resource bricolage in Model 8. The interaction term of resource bricolage and environmental dynamism had a significant effect on career growth (β = 0.155, *p* = 0.001), thus warranting the further Model 9.

On the basis of Model 8, Model 9 adds potential moderators to career growth. The results show that career growth had a significant effect on urban integration (β = 0.272, *p* < 0.001), and that the moderating effect of resource bricolage and environmental dynamism (β = 0.097, *p* = 0.062) was significant. This indicates a moderating effect of resource bricolage and environmental dynamism on urban integration through career growth, thus supporting Hypothesis 6.

## Discussion

As an important indicator of citizenization, the urban integration of new-generation migrant workers reflects the meaning and quality of urbanization. Previous studies have approached this topic from a macro perspective at the policy level, whereas relatively few studies have taken a micro perspective. This empirical study takes new-generation migrant workers as the research object and examines resource bricolage rather than traditional single resource utilization. The findings reveal a significant relationship between resource bricolage and the career growth and urban integration of new-generation migrant workers, and highlight the role of career growth and environmental dynamism variables in this mechanism. The four main findings are discussed below.

(1) Resource bricolage was found to have had a significant positive impact on the career growth and urban integration of new-generation migrant workers. This finding suggests that resource bricolage is more than the ability of new-generation migrant workers to obtain urban employment and survive in the city, as it also represents the individual’s developmental capabilities in the process of citizenization. Although the urban resources of new-generation migrant workers are limited, they can promote their career growth and urban integration through resource bricolage.

Early studies have found that resource bricolage had an impact on the career success and performance of entrepreneurs, such as farmers and family farms [19,20,22]. This study extends the research object to new-generation migrant workers and finds that resource bricolage also promotes the growth of new-generation migrant workers.

In addition, this quantitative finding to some extent validates previous qualitative research in this field [21]. It appears that a simple bricolage of single resources can only meet the primary growth needs of migrant workers, with career success dependent on the mixed bricolage of multiple types of resources; this leads to a demand for greater resource bricolage abilities in migrant workers. This study also further explores the relationship between resource bricolage and urban integration, which is a non-entrepreneurial performance variable, and provides strong evidence for understanding the relationship between resource bricolage and urban integration of new-generation migrant workers at the micro level.

(2) Career growth was found to play a partial mediating role in the relationship between resource bricolage and urban integration. This suggests that the transformation effect of resource bricolage on urban integration is not achieved overnight but requires a certain transformation process. Specifically, the effect of resource bricolage on urban integration is gradually realized through continuous career growth. Therefore, we need to pay attention not only to the urban migration of new-generation migrant workers but also to their survival in the city and, more importantly, their career growth. For new-generation migrant workers, entering cities for work is a prerequisite for citizenization, and career growth is the key factor in their urban integration and citizenization.

This result shows that career growth is an important condition that promotes the urban integration of new-generation migrant workers, which is in line with early research findings that employment promotes citizenization [56]. The proposition that career growth can promote citizenization has been verified, and this finding further extends the scope of this effect from the pre-citizenization stage to the citizenization stage.

Another way to understand this mechanism is that in the process of citizenization, new-generation migrant workers have two goals: career growth and urban integration. The career growth of new-generation migrant workers is the stage goal of their urban development [32], but the real goal of their urban work and life is to obtain “career happiness” and “life happiness,” and to realize the life goals of a “house purchase,” their “children’s education,” and “household registration” [56]. Fundamentally, the stage goal is the means to help realize the substantial goal of citizenization. Therefore, the role of career growth in the formation mechanism of new-generation migrant workers’ urban integration should not be ignored. Career growth and urban integration not only are parallel processes but also have a causal relationship.

(3) Environmental dynamism was found to have positively moderated the relationship between resource bricolage and urban integration. This means that as the external environment becomes more dynamic, new-generation migrant workers’ need to integrate resources becomes more extensive and powerful, as this is one of the conditions needed to ensure their integration into the city.

Environmental dynamism is closely related to resource bricolage. When facing the pressures of a dynamic external environment, new-generation migrant workers will self-intervene and promote their own development through the optimization of resources and capabilities [34,35]. In this context, resource bricolage takes the meaning of an adaptive integration to deal with the dynamic environment.

This study also found that the external environment presents both challenges and opportunities to new-generation migrant workers. Although environmental dynamism may not always be desirable, it creates certain environmental opportunities for the resource bricolage of new-generation migrant workers. Specifically, environmental dynamism promotes the combination of resources, thus presenting external incentives for resource bricolage.

(4) This study found a moderating effect of the resource bricolage of new-generation migrant workers and environmental dynamism on urban integration through the mediation effect of career growth. This suggests that in a dynamic environment, whether resource bricolage has a practical impact on urban integration depends on the transformational role of career growth. Given the conditions of environmental dynamism, if new-generation migrant workers lack support for career growth, realizing the effects of resource bricolage on urban integration will be difficult. This finding further verifies the role of career growth in the citizenization of new-generation migrant workers [56].

There are some limitations to this study. First, only new-generation migrant workers working in the Yangtze River Delta region were selected to participate. Although the Yangtze River Delta is a major region for the inward migration of workers in China, regional characteristics may reduce the universality of the research results to a certain extent. In view of this, future studies could expand the geographic scope of the sample to the Pearl River Delta and to some inland provinces of China to verify the results. Second, in-depth interviews might help to increase the understanding of typical cases of resource bricolage among new-generation migrant workers, and the types and processes of this bricolage could be expanded upon and categorized using qualitative research methods; such an approach would deepen the theory and understanding of the resource bricolage of new-generation migrant workers.

## Conclusions

The results of this study show that resource bricolage can promote the career growth of new-generation migrant workers and further promote their city integration, and that environmental dynamism represents the external conditions that can promote the resource bricolage of new-generation migrant workers. In summary, resource bricolage plays a key role in this mechanism. For new-generation migrant workers, resource bricolage represents not only a resource integration ability but also a means for better adapting to an unfair urban competitive environment and achieving growth. Returning to the realization of the basic values of fairness and justice in urbanization in China, new-generation migrant workers need to be assisted materially and psychologically by the government, society, enterprises, and communities. In particular, supplementing the lack of resources in terms of skills and knowledge can promote the realization of urban integration and citizenization among new-generation migrant workers.

## Supporting information

S1 Data (XLSX)
